# Glucose dysregulation in pre-clinical Alzheimer’s disease

**DOI:** 10.18632/aging.102146

**Published:** 2019-08-04

**Authors:** Niamh M. C. Connolly, Pierre Theurey, Paola Pizzo

**Affiliations:** 1 Centre for Systems Medicine and Dept. of Physiology and Medical Physics, Royal College of Surgeons in Ireland, Dublin 2, Ireland; 2 Department of Biomedical Sciences, University of Padua, Padua, Italy; 3 Neuroscience Institute - Italian National Research Council (CNR), Padua, Italy

**Keywords:** Alzheimer’s disease, glucose metabolism, mitochondria, systems biology, bioenergetics

Alzheimer’s disease (AD) is the most common cause of age-related dementia and neurodegenerative disease [1]. Despite the impact on public health and the economic burden in ageing societies, no disease-modifying therapies exist, and AD remains incurable. AD pathology can begin long before the appearance of outward clinical symptoms [1–3], and the well-publicised failures of clinical trials targeting symptomatic AD highlight the need for pre-symptomatic biomarkers and therapies. Although theories abound for the underlying mechanisms of neurodegeneration in AD, studies are commonly performed in post-mortem AD brains or in models where pathology is already apparent. Elucidation of pathology initiation and progression at the molecular level requires the use of appropriate pre-symptomatic AD models and innovative analytical approaches. Using experimental and computational techniques, we recently demonstrated an impairment of glucose metabolism in primary neurons from a double transgenic mouse model of AD (PS2_N141I_APP_swe_/B6.152H), and showed that this impairment affects NADH-mediated mitochondrial bioenergetics while mitochondrial integrity remains intact [4]. Interestingly, glucose hypo-metabolism occurs in clinical AD and correlates with the severity of cognitive decline [1,5]. Moreover, glucose metabolism abnormalities can be measured before the onset of cognitive impairment, may begin years or even decades before the onset of clinical symptoms, and are reliable indicators of disease progression [1,3]. Although ^18^FDG-PET imaging studies in AD animal models are thus far inconsistent [6], our study demonstrated that glucose metabolism defects are detectable *in vitro* prior to the appearance of Amyloid beta (Aβ) pathology or behavioural phenotypes, and highlighted the utility of an inter-disciplinary approach [4].

The consequences of glucose hypo-metabolism are manifold, most of which have been reported in AD. Reduced cytosolic NADH production and carbon fluxes to mitochondria contribute to impaired mitochondrial function and potential bioenergetic crisis involving reduced ATP production, increased ROS levels and oxidative stress, calcium de-regulation, synaptic activity disruption, and neurodegeneration. The pentose phosphate pathway, other biosynthetic pathways, glycosylation, and mitochondrial movement can also be affected. Even in the absence of overt bioenergetic crisis, glucose dysregulation may hamper the neuron’s ability to withstand insults such as excessive cytosolic calcium levels and excitotoxic stress [7]. Indeed, we recently observed that primary hippocampal neurons from the B6.152H model above are more sensitive to KCl-induced calcium overload (Rigotto G. and Basso E., unpublished research). Impaired energy metabolism can also contribute to Aβ accumulation and tau hyper-phosphorylation [1]. Interestingly, neurons with longer axons may be more vulnerable in AD due to increased energy requirements, spatially heterogeneous distribution of glucose uptake and processing machinery, and reliance on axonal transport. Finally, reduced carbon metabolism may induce the catabolism of alternative lipids and amino acids [3] and alter the synthesis of some amino acid-derived neurotransmitters. Thus, while metabolic flexibility may enable neurons to successfully overcome short-term stress conditions, chronic neuronal alterations could be detrimental. Importantly, altered composition of cell metabolites in the cytosol and extracellular milieu could provide early biomarkers of pathology in peripheral fluids. Indeed, fluctuations in the circulating levels of key metabolites linked to mitochondrial activity have been reported in AD [3].

The causes of pathological glucose hypo-metabolism in AD are likely multi-factorial. Glycolytic enzyme activities have been found to be reduced in AD, although inconsistently [4,5]. Calcium signalling is an important regulator of glucose metabolism, and its dysregulation occurs in AD and was measured in the B6.152H model [8]. ^18^FDG-PET studies suggest that decreased glucose uptake is an early feature of AD pathogenesis - depletion of neuronal glucose transporters has been reported [1], although we did not measure this in our system [4]. Interestingly brain insulin resistance, which occurs in AD, can also lead to reduced neuronal glucose uptake, and type 2 diabetes is a risk factor for developing AD [1,2,5]. Other mechanisms may include oxidative stress, which can damage glycolytic enzymes and other metabolic systems thereby fuelling a toxic cycle [5], or Wnt signalling, which regulates glucose uptake and utilisation [4]. Although glucose hypo-metabolism has been measured prior to the observation of Aβ pathology [1,4], early or exacerbating effects of Aβ cannot be discounted. Multiple complementary therapeutic strategies will therefore be required to alleviate or bypass pathological effects of glucose hypo-metabolism, including molecular interventions, alternative energy sources, or antioxidants (protective effects have been demonstrated in pre-clinical models, but clinical trials have been disappointing [5]). Some may be accessible through lifestyle interventions such as diet and exercise (see for example http://wwfingers.com). Insulin sensitizers, such as metformin, are being investigated as AD therapeutics, and intra-nasal insulin moderately improved FDG-PET metabolism and cognitive function in a pilot trial for people with AD [2].

In summary, AD is the most common cause of age-related dementia and is consistently accompanied by glucose hypo-metabolism that precedes the onset of clinical symptoms, providing potential for early diagnosis and novel therapeutic interventions. Innovative inter-disciplinary research utilising appropriate pre-clinical models is required to further elucidate the molecular mechanisms, cross-talk with Aβ and tau pathology, and translatable approaches targeted to the early stages of AD. Given that glucose hypo‑metabolism has also been measured in other neurodegenerative diseases, we are excited to watch this field progress.
